# Information–Entropy Analysis of Stellar Evolutionary Stages with Application to FS CMa Objects

**DOI:** 10.3390/e27111106

**Published:** 2025-10-26

**Authors:** Zeinulla Zhanabaev, Aigerim Akniyazova, Yeskendyr Ashimov

**Affiliations:** Faculty of Physics and Technology, Al-Farabi Kazakh National University, Al-Farabi Ave., 71, Almaty 050040, Kazakhstan; zhanabayev.zeynulla@gmail.com (Z.Z.); aigerimakniyazova@gmail.com (A.A.)

**Keywords:** information entropy, statistical physics, stellar evolution, FS CMa stars

## Abstract

Theoretical foundations are presented for the application of information–entropy methods from statistical physics to the determination of stellar evolutionary stages. A balance equation involving normalized conditional information and entropy is proposed. The conditional information is defined as the difference between the entropy of the phase space and the conditional probability entropy. A correspondence is demonstrated between theoretical predictions and observational data from stellar emission spectra with respect to their evolutionary classification. The proposed methodology is further applied to the analysis of complex FS CMa-type objects, which exhibit dusty and gaseous structures with components at different evolutionary stages. In this context, the conditional information derived from asymmetric spectral lines is shown to be consistent with the theoretical criteria for the evolutionary status of single, binary, and unclassified stars.

## 1. Introduction

Stellar evolution is the process that describes the physical changes stars undergo throughout their lifetimes. This evolutionary process begins with the formation of a star from interstellar gas and dust and ends in various final stages—such as white dwarfs, neutron stars, or black holes—depending on the initial stellar mass. The study of stellar evolution represents a cornerstone of modern astrophysics, as stars are the primary sources of light and chemical elements in the Universe, shaping its structure and dynamics. Stars form within giant molecular clouds, where gravitational instabilities trigger the collapse of dense regions [1]. This collapse leads to the formation of protostars, which subsequently evolve through the phase of hydrogen fusion and enter the main sequence stage [2]. During this phase, a star maintains hydrostatic equilibrium, balancing the outward radiation pressure generated by nuclear fusion against gravitational contraction. For solar-mass stars, this phase lasts approximately 10 billion years. More massive stars, however, consume their nuclear fuel at a much higher rate, resulting in significantly shorter main-sequence lifetimes [3]. Once hydrogen is exhausted in the stellar core, the star evolves into the post-main-sequence phase. Low- and intermediate-mass stars (up to 8 solar masses) expand into red giants and eventually shed their outer envelopes, forming planetary nebulae with a white dwarf remnant at the center. In contrast, high-mass stars may end their lives in supernova explosions, leaving behind neutron stars or black holes [4,5]. For many stars, the evolutionary pathway becomes substantially more complex if they reside in binary or multiple systems. Gravitational interactions between the components can lead to mass transfer, the formation of accretion disks, and profound alterations in the evolutionary pathways of both stars. Such interactions may give rise to unique astrophysical phenomena, including cataclysmic variables, X-ray binaries, and other compact and exotic stellar remnants. A binary star system consists of two stars gravitationally bound and orbiting a common center of mass. These systems serve as natural laboratories for studying stellar physics, as they provide critical insights into mass and angular momentum exchange, as well as the role of gravitational interactions in shaping various stages of stellar evolution [6,7,8]. In addition, the study of FS CMa-type binary stars has recently gained considerable relevance. FS CMa-type stars are characterized by strong emission lines in their spectra, especially in the hydrogen Balmer series, and are hot stars with effective temperatures ranging from 10,000 to 30,000 K. Stars with the B[e] phenomenon are divided into the following subgroups: Herbig Ae/Be stars located before the main sequence (HAeBe); symbiotic binary systems, consisting of a cool giant and a white dwarf or neutron star (symB[e]); compact planetary nebulae (cPNB[e]); supergiants (sgB[e]); unclassified (unclB[e]). Based on a long-term study of the “unclassified” objects, Miroshnichenko A.S. (2007) proposed [9,10] that most of the “unclassified” objects are neither Herbig Ae/Be (HAeBe) pre-main sequence stars nor supergiants, but binary systems, and classified them into a new group of FS CMa objects in honor of the prototype object HD 45677 (designated FS CMa in the General Catalog of Variable Stars). These stars are a special group of objects that have attracted considerable attention from researchers due to their unique characteristics and complex evolutionary processes. Recent studies have examined in detail the spectral and photometric properties of FS CMa stars, which are characterized by the presence of intense emission lines and an infrared excess due to circumstellar dust and gas [11]. These studies have shown that FS CMa stars are predominantly binary systems in which one component is a pre-main-sequence (PMS) star, while the other is either on the main sequence (MS) or represents a compact post-MS object. The diversity of stellar properties necessitates a classification scheme based on their observable characteristics and evolutionary stages. The stellar spectrum represents the most informative parameter for classification. The well-established Hertzsprung–Russell diagram, relating stellar luminosity to spectral class (O, B, A, F, G, K, M, and L in order of decreasing effective temperature) 6 reveals multiparametric and nonlinear dependence. Because of this, approaches to dividing each class into 10 subclasses were proposed. If we are interested in the time evolution of each star, then it is obvious that it is necessary to use the probabilistic regularities in the behavior of spectral lines. The work is devoted to the information–entropy analysis of the evolution of stars of different spectral classes. Section 1 presents the characteristics (temperature, mass, and luminosity) and individual types of evolution according to the criteria of conditional information I(Y|X) without taking into account the fluctuations of the information itself (Table 1). Section 2 describes in detail that the theoretical basis for defining conditional information is defined through the difference in entropies of the phase space and the constraints, expressed as the time derivative of the signal power spectrum intensity. Section 3 presents algorithms taking into account the change in the power spectrum due to fluctuations in the wavelength of individual atomic lines and the results of application (Table 2) to increase the accuracy of the data (Table 1). We have listed the main physical processes of stellar evolution occurring at the nuclear, atomic, and molecular level and in a wide range of radiation spectra. These processes are caused by nonlinear interactions of elements of the stellar environment and stellar components with different evolutionary statuses (FS CMa objects). Nonlinear interactions commonly result in bifurcation, chaotic dynamics, and stochastic phenomena, which can be described within the framework of modern statistical physics. Therefore, we consider it possible to use, in addition to existing methods, information–entropy approaches for determining the evolutionary status of stars.

## 2. Methods

### 2.1. Observational Data on Stellar Properties

To assess the applicability of the proposed methodology, we selected a sample of stars with well-established evolutionary statuses and analyzed their fundamental physical parameters. The selection was based on their locations across different evolutionary stages on the Hertzsprung–Russell diagram. For clarity, and in accordance with the theoretical model based on the conditional information measure $\tilde{I}\left(Y|X,\lambda_{0}\right)$, we categorize the stars into three evolutionary types: I—pre-main-sequence (PMS) stars; II—stars main-sequence (MS) stars; III—evolved, post-main-sequence (post-MS) stars. This classification is derived from the values $\tilde{I}\left(Y|X,\lambda_{0}\right)$ without considering fluctuations in the information content.

The stellar data used in this study (Table 1) were obtained from the PolarBase archive, which compiles high-resolution stellar spectropolarimetric observations. These data were obtained with instruments mounted on the Bernard Lyot Telescope (TBL) and the Canada–France–Hawaii Telescope (CFHT) [35].

The spectra of these stars cover a wide range of wavelengths, from the ultraviolet to the infrared. For the purposes of our analysis, the optical range is most important, since, for the application of the information–entropy method, we primarily rely on the stronger profiles of the HeI and Hα lines, among others.

FS CMa stars are a relatively recent and still poorly understood class of stellar objects, first recognized as a distinct class in 2007 [10]. These stars exhibit a distinctive emission spectrum characterized by their complex and not yet fully understood nature. The evolutionary status of FS CMa stars remains a matter of debate. Some studies suggest that they may be young stars at the protostar stage or stars in late stages of evolution, such as post-AGB stars or interacting binaries. A more detailed discussion of this class is provided in Section 4.

### 2.2. Information–Entropy Analysis

The concepts of information and information entropy are rooted in the classical works of C. Shannon on probability theory and communication. In physical terms, proper information $I\left(x_{i}\right)$ represents a measure of certainty associated with a physical quantity $X=\left\{x_{i}\right\}$ with discrete values $\left[x_{i}\right]$. Information entropy H(X) (hereinafter preferred simply as “entropy”) corresponds to the average value *X*, serving as a measure of uncertainty:

(1) $$I\left(\left[x_{i}\right]\right)=-\ln p\left(\left[x_{i}\right]\right)$$

(2) $$H\left(X\right)=-\sum_{i}p\left[x_{i}\right]\ln p\left(\left[x_{i}\right]\right),$$

where $p\left[x_{i}\right]$ denotes the probability of occurrence of the discrete values $\left[x_{i}\right]$. In applied analysis, discrete quantities $\left[x_{ij}\right]$ are used, since defining entropy by integrating over the probability density, where f(x)(dp(x)=f(x)dx) and ln(f(x)dx), introduces an ambiguity as dx→0.

Physical processes are typically characterized by correlations between at least two characteristic quantities, $X\left\{x_{i}\right\},Y\left\{y_{i}\right\}$. In such cases, the information is determined by the interaction between the values of the elements of the sets X,Y. In communication and information theory, Shannon’s mutual information I(Y/X) [36,37] is widely used:

(3) I(Y∣X)=H(X)−H(Y∣X),

where conditional entropy is represented as follows:

(4) $$H\left(Y|X\right)=-\sum_{i=1}^{N}\sum_{j=1}^{M}p\left(x\left[i\right],y\left[j\right]\right)\log_{2}p\left(y\left[j\right]|x\left[i\right]\right),$$

The conditional probability is determined by Bayes’ theorem:

(5) p(y[j]∣x[i])=p(y[j],x[i])∣p(x[i])

In the literature, mutual information is also expressed through the elements of the sets *X* and *Y* $I\left(x_{i};y_{j}\right)$ using the notation $I\left(x_{i};y_{j}\right)$.

Expression (3), by applying Formula (5), can be rewritten in an explicitly symmetric form.

(6) $$I\left(Y|X\right)=I\left(X|Y\right)=\sum_{i}\sum_{j}p\left(\left[x_{i}\right],p\left[y_{j}\right]\right)\log_{2}\left(\frac{p\left(\left[x_{i}\right],\left[y_{j}\right]\right)}{p\left(\left[x_{i}\right],\left[y_{j}\right]\right)}\right)\geq 0$$

If there is no correlation between *X* and $Y\left(p\left(\left[x_{i}\right],\left[y_{j}\right]\right)=p\left[x_{i}\right]p\left[y_{j}\right]\right)$, the equality holds.

Mutual information (3) is a symmetric measure of correlation between X⇄Y and can be applied only to known signals *X* (transmitted) and *Y* (received). However, the relationships X=X(Y), Y=Y(X) may be irreversible or nonlinear.

For astrophysical applications we employ an asymmetric model of conditional information I(Y∣X), which is defined as the difference between the entropy of the joint ensemble H(X,Y) and the conditional entropy H(Y∣X):

(7) I(Y∣X)=H(X,Y)−H(Y∣X)

From Formula (7) it follows that the conditional information is asymmetric, i.e., I(Y∣X)≠I(X∣Y). This asymmetry arises because, although the joint entropy is additive and symmetric (H(Y,X)=H(X,Y)), the conditional entropies differ: H(Y∣X)≠H(X∣Y).

In the context of our analysis, the dependence Y=Y(X), describing the intensities of astrophysical signal spectra *X*, is nonlinear and irreversible.

Furthermore, by applying Bayes’ formula to the probability distributions and the corresponding entropy functions, one can relate the conditional and joint entropies in a consistent probabilistic framework.

(8) $$p\left(\left[x_{i}\right],\left[y_{j}\right]\right)=p\left(\left[x_{i}\right]|p\left[y_{j}\right]\right)p\left(\left[x_{i}\right]\right),\;H\left(Y,X\right)=H\left(X\right)+H\left(Y|X\right),$$

The proposed measure, I(Y∣X), can be regarded as the conditional entropy of the signal H(X) [37]; however, this interpretation does not, in general, reflect the physical meaning of I(Y∣X). The reduction in the total ensemble entropy H(X,Y) by the entropy of its component H(Y∣X) characterizes the emergence of order-information. The quantity H(X) itself is derived from $p(\left[x_{i}\right])$, which depends on the joint probability distribution $p(\left[x_{i}\right],\left[y_{j}\right])$ that defines H(X,Y).

Establishing the relationship between conditional information and entropy is therefore crucial for describing the evolutionary status of stars and for analyzing transitional and bifurcation phenomena, as it quantitatively captures the degree of order and chaos in the system.

By normalizing Formula (7) with respect to H(X,Y), we derive a unique conservation relation between the normalized conditional information and entropy, which describes the transitions from order $(\tilde{I})↔$ to chaos ($\tilde{H}$) [37].

(9) $$\tilde{I}\left(Y|X\right)+\tilde{H}\left(Y|X\right)=1,\;\tilde{H}=H\left(Y|X\right)/H\left(X,Y\right)\;\tilde{I}=I\left(Y|X\right)/H\left(X,Y\right)$$

In Formula (9), we assume Y=Y(X), that is, the condition *Y* is determined from the signal as its first derivative; in other words, we use the phase portrait representation.

The conditional function Y=Y(X) can be defined in various forms, reflecting the specific characteristics of the process. For processes involving acceleration, Y(X) is obtained through the second derivative with respect to time X(t) [37]. The contribution of standard noise processes can also be taken into account, among other factors.

Formula (9) alternatively separates the normalized conditional information and entropy with respect to their equilibrium values of 1/2. For the analysis of complex systems (such as binary stars and FS CMa-type objects), it is necessary to quantitatively distinguish the conditional information $\tilde{I}\left(Y|X\right)$ in Formula (9).

Let us now introduce the well-known concept of proper information I=I(P), which characterizes the randomness of the information itself I(P) with probability

(10) $$P\left(I_{i}\right)=e^{-I_{i}}$$

in accordance with Formula (1). The self-similarity (or scale invariance) of information implies the existence of a fixed point [38]:

(11) $$e^{-I_{*}}=I_{*}$$

Using iterative or graphical methods, we obtain a self-similar and stable value of self-information $I_{*}$ = 0.576.

The fixed value of the entropy of self-information is defined as

(12) $$H\left(I_{*i}\right)=\sum_{i}e^{-I_{*i}}*I_{*i}=I_{*i}$$

Formula (12) does not include the logarithm of the product of the information probability density and its increment; therefore, through integration, we obtain the following result:

(13) $$H\left(I\right)=\int_{I}^{\infty }e^{-I}IdI=\left(I+1\right)e^{-I}$$

The condition of scale invariance in Formula (13) yields $H\left(I_{*}\right)=I_{*}$ with $I_{*,H}=0.806$. The criteria $I_{*}=0.567$ and $I_{H}=0.806$ describe the modes of self-similarity (scale invariance) and represent a generalization of the well-known “golden section of the dynamic measure” of Fibonacci to probabilistic phenomena. Indeed, from Formulas (12) and (13), for I≪1, it follows that $I_{*}=0.567$, since for I<1 the expansion $e^{-I}\approx 1-I$ leads to the Fibonacci-type equation $I^{2}+I-1=0$, whose solution is I=0.618.

To apply Formula (9) and the self-similarity information criteria $I_{*}\left(p\right)$ and $H\left(I_{*}\right)$ to the processing of complex signals, such as those of FS CMa-type objects, we define the dependence of the quantities $\tilde{I}\left(Y|X\right)$ and $\tilde{H}\left(Y|X\right)$ on their ratio $\tilde{I}\left(Y|X\right)/\tilde{H}\left(Y|X\right)$. This dependence has a clear physical interpretation. From Formula (9) it follows that

(14) $$\tilde{I}\left(Y|X\right)/\tilde{H}\left(Y|X\right)=1/\tilde{H}\left(Y|X\right)-1$$

The left-hand side of Formula (14) represents the ratio of the measures of certainty and uncertainty (analogous to the signal-to-noise ratio, SNR), while the right-hand side provides a simpler computational result derived from Formula (9).

**Figure 1:**
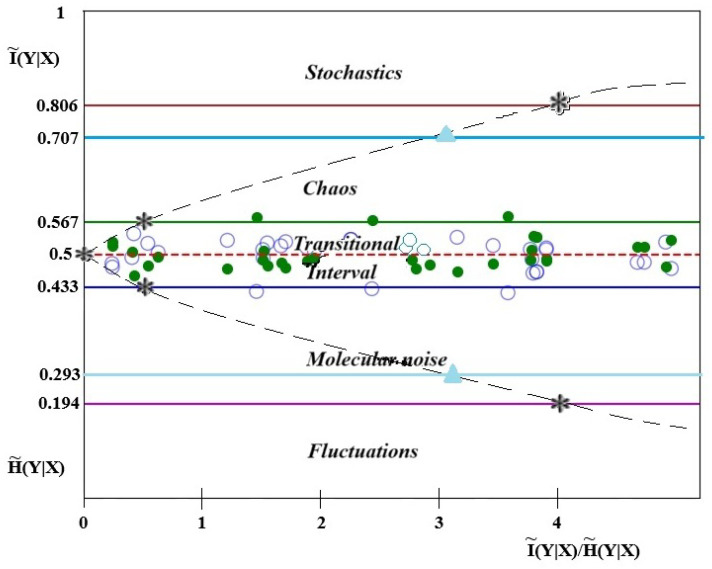
Change in conditional information and entropy characteristics by their ratio (information analogue of the signal-to-noise ratio—SNR). As an example, in the transition region, the values of self-similarity (eigensymmetry of functions) of information (∘) and entropy (•) are given for a status III star.

For a low value of self-similarity, $H\left(I_{*}\right)=1-0.806=0.194$, Formula (14) yields $(\tilde{I}\left(Y|X\right)/\tilde{H}\left(Y|X\right)\approx 4$. In the context of classifying the evolutionary status of stars, Figure 1 may provide useful insight.

The terms chaos and stochastics are discussed in detail in the literature on nonlinear oscillations and dynamical chaos, both from theoretical and experimental perspectives. Briefly, stochastics refers to a fluctuating, purely statistical phenomenon, whereas chaos represents a structured, nonlinear dynamical behavior that arises without an external source of fluctuations, yet can be described through statistical regularities. In particular, we note the study [39,40], which presents both theoretical and experimental results on this subject.

## 3. Main Results

### 3.1. Results of Information–Entropy Analysis

For clarity, before applying Formula (9), phase portraits based on spectral line intensities were constructed. A phase portrait is a standard tool in dynamical systems theory used to visualize the temporal behavior of a system. To construct a phase portrait, the function X(t) is taken, and its first derivative Y(t) with respect to time is calculated from the line intensity in order to identify evolution types I, II, and III. The (X,Y) plane is divided into equal cells along both the *X* and *Y* axes, and the probabilities of points falling within each cell are determined as $p\left(\left[x_{i}\right],\left[y_{j}\right]\right)$. According to Formula (4), the conditional entropy is then calculated.

**Figure 2:**
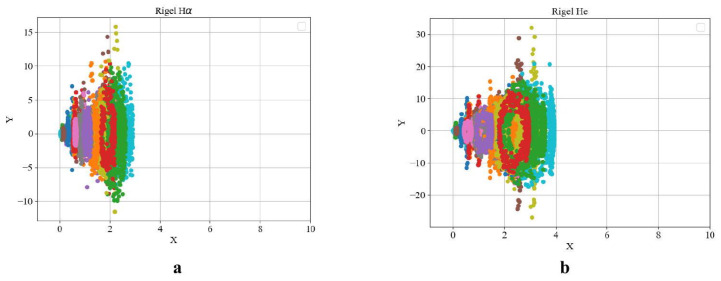
Phase portraits for the star Rigel with evolution status II based on spectral lines (λ6560–6566) (**a**) and He(λ5875) (**b**). The HαX-axis shows the intensity of the spectrum lines, and *I* its derivative with respect to the τY-axis.

Figure 2 reveal the following general patterns, which are supported by additional examples. The phase portraits of the key elements participating in thermonuclear reactions—namely hydrogen (Hα) and helium—exhibit qualitatively similar behavior.

**Figure 3:**
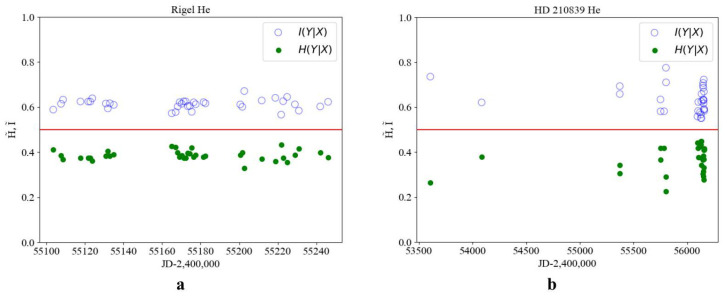
Conditional information $\tilde{I}$ (light dots) and entropy $\tilde{H}$ (dark dots) for the star Rigel (β Orionis), evolution status II (**a**), and for the star HD 210839 evolution status I (**b**) HJD, for the He line profile (λ5875).

In young stars with evolutionary status I, such as HD 210839, the values of conditional information $\tilde{I}$ and entropy $\tilde{H}$ are separated relative to the theoretical equilibrium value of 1/2. The measure of order exceeds that of chaos ($\tilde{I}>\tilde{H}$), and no order–chaos transition is observed. The use of time-dependent sampling does not alter the relative positions of $\tilde{I}$ and $\tilde{H}$ with respect to 1/2 as presented in Figure 3b.

**Figure 4:**
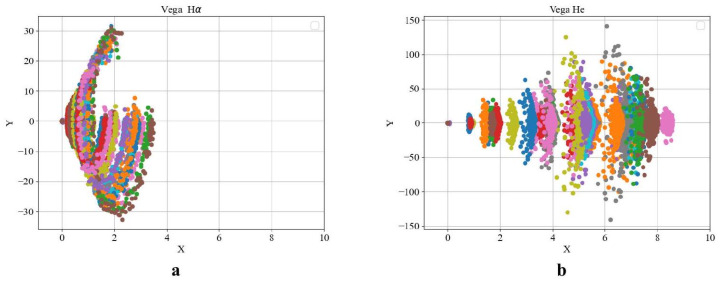
Phase portrait for the star Vega with evolution status II based on spectral lines (**a**) Hα(λ6561–6563) and the (**b**) He line profile (λ5875).

**Figure 5:**
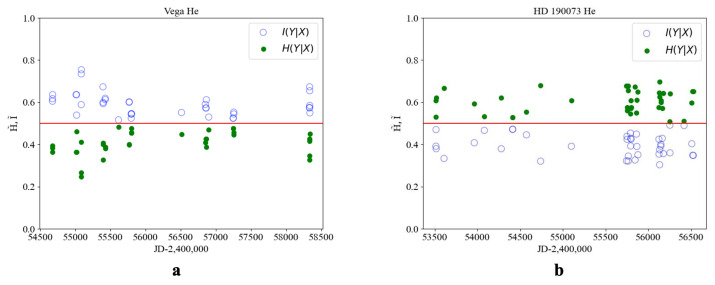
Conditional information $\tilde{I}$ (light dots) and entropy $\tilde{H}$ (dark dots) for the star Vega (**a**) and for the star HD190073 with evolution status II (**b**) HJD for the He line profile (λ5875).

A stratification can be observed in the phase portrait of the Hα line, corresponding to an earlier evolutionary state, whereas the phase portrait of helium (He) reflects a more integrated structure associated with a later stage as presented in Figure 4. HD 190073 is a relatively stable star in the middle of its evolutionary path. Elemental interactions are observed, resulting in two transition events out of 20 cases (Figure 5b).

Significant changes in the phase portraits, as well as in the conditional information and entropy, are observed for stars with evolutionary status III. At this final evolutionary stage, the intensities of the energy-release components Hα, He are weak; however, due to possible accretion processes and stellar winds from the surrounding environment, occasional bursts in the hydrogen line Hα are detected.

**Figure 6:**
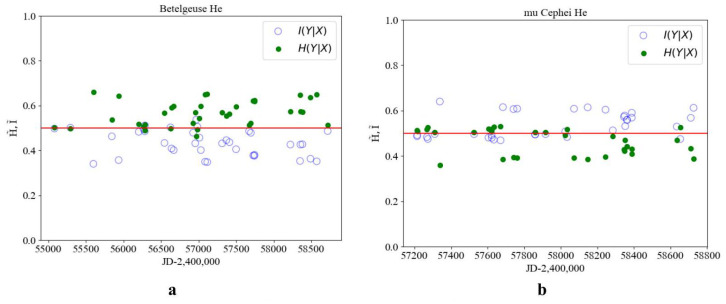
Conditional information $\tilde{I}$ (light dots) and entropy $\tilde{H}$ (dark dots) for the star Betelgeuse (**a**) and for the star M Cephei, evolution status III (**b**) HJD for the He line profile (λ5875).

μ Cephei is a star in the final stage of its evolution and is therefore enriched in heavy elements. As a result, entropy–information transitions were observed at 12 points out of 35 cases (Figure 6b).

**Figure 7:**
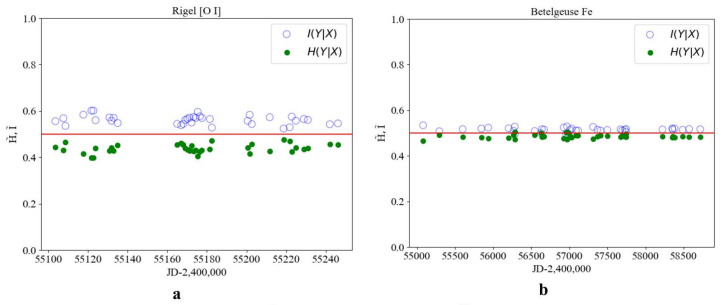
Conditional information $\tilde{I}$ (light dots) and entropy $\tilde{H}$ (dark dots) for the star Rigel and Betelgeuse with evolution status II and III: (**a**) change in from HJD, Rigel, for the spectral line [OI](λ7775); (**b**) change according to HJD, Betelgeuse, for the spectral line [FeI] (λ5014) evolution status III.

To further confirm the identified regularities, the conditional information and entropy were calculated for the [OI] spectral line of the oxygen triplet (λ7775) in the star Rigel and for the Fe spectral line (λ5014) in Betelgeuse (Figure 7). As expected, the values of $\tilde{I}$ and $\tilde{H}$ for the lines and spectra of heavy elements are close near the transition regions; however, no distinct transitions are observed.

The information–entropy analysis of spectral line profiles for stars at different evolutionary stages reveals the following regularities. For stars with evolution status I, no contact between information and entropy values is observed, and thus the order–chaos transition does not occur. Status II stars exhibit intersections of information and entropy values near the theoretical line at 0.5, with one transition detected among 20 points. Status III stars show information and entropy transitions at an average of 12 out of 35 points, indicating their transition toward a chaotic state in accordance with the theoretical result $I_{*}=$0.433÷0.567, where 0.433=1−0.567. If the order parameter value is less than 0.433, the classification can be made according to Figure 1.

### 3.2. Results of Information–Entropy Analysis of FS CMa Type Stars

FS CMa stars represent a distinct and relatively poorly studied group of objects. They differ from other stellar classes due to their unique spectral characteristics and evolutionary peculiarities. The nature and origin of FS CMa stars were investigated by Miroshnichenko A. S. [10,41].

**Figure 8:**
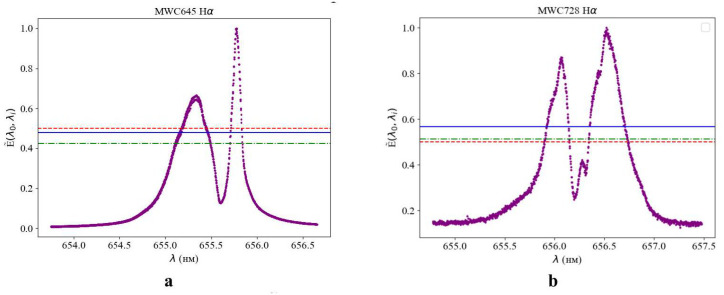
Averaging the intensity $\tilde{E}\left(\lambda_{0},\lambda_{i}\right)$ over discrete values of $\lambda_{i}$ spectral lines Hα for FS CMa type stars taking into account the profile asymmetry (**a**) MWC645 and (**b**) MWC728.

These features make FS CMa stars important targets for studying transitional phases of stellar evolution. A new algorithm, shown in Figure 8, has been introduced to describe the complex interaction processes between the stellar components.

To describe the complex interaction processes between stellar components, a new algorithm was applied, as illustrated in Figure 8.

For complex stellar systems with interacting components, the normalized power spectrum values were calculated using Formula (15).

(15) $$\tilde{E}\left(\lambda_{0},\lambda_{i}\right)=\frac{\sum_{i=1}^{N}{\tilde{E}}_{l}\left(\lambda_{0},\lambda_{i}\right)\Delta \lambda_{i}}{\sum_{i=1}^{N}\Delta \lambda_{i}}$$

This expression (a histogram of discrete values) provides a more accurate description of the state of a stellar system with an asymmetric spectral profile. The conventional method of using the “spectral half-width” to characterize the attenuation decrement as a function of measurement frequency is applicable mainly to symmetric, bell-shaped spectral profiles that follow Gaussian or Lorentzian (Cauchy) probability density distributions. In contrast, the different values of $\tilde{E}\left(\lambda_{0},\lambda_{i}\right)$ quantitatively characterize the state of the system by describing physical criteria $\tilde{I}\left(Y|X\right)$ and $\tilde{H}\left(Y|X\right)$ (Figure 8).

The dotted lines indicate the reference theoretical level of 0.5, while the dash–dotted lines correspond to the values calculated using Formula (9) within the framework of information–entropy analysis. The solid lines were obtained using Formula (15), which accounts for the asymmetry of the spectral profile when averaging the discrete values ${\tilde{E}}_{l}\left(\lambda_{0},\lambda_{i}\right)$. The consistency between the results obtained by different methods confirms the reliability of the determined evolutionary status of the stars presented in Table 2.

In Figure 8, the dashed lines represent the possible positions of a binary star system in a locally isotropic space. The calculation of the normalized intensity $\tilde{E}\left(\lambda_{0},\lambda_{i}\right)$ using Formula (15) makes it possible to distinguish between a physical (nonrandom) pair and single stars. With an appropriate choice of $\Delta \lambda_{i}$ for the intense spectral line of the emitting element, $\tilde{E}\left(\lambda_{0},\lambda_{i}\right)$ can also be used to identify eclipsing binaries (even with relatively short observation periods), multiple systems, and stellar clusters.

These results indicate that by accounting for the inherent randomness of the information extracted from the signal, one can determine self-similar (scale-invariant) probabilities of detection and the average information (entropy) value of the signal (Formula (9)). The diversity of fluctuation phenomena observed in nonlinear systems of various origins can be generalized within the framework of the period-doubling bifurcation and Feigenbaum scale invariance, as well as the fluctuation–dissipation theorem, as described in well-known studies [39].

**Figure 9:**
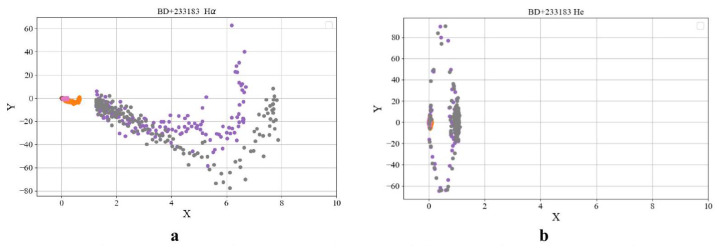
Phase portrait for the star BD+233183 with the B[e] phenomenon based on spectral lines Hα(λ6563–6566) (**a**) and He(λ5875) (**b**).

For FS CMa-type stars, phase portraits of spectral line intensities were additionally constructed (Figure 9). The phase space of FS CMa stars corresponds to evolutionary types II and III. The more intense spectral lines X≫7 exhibit faster temporal variations (Y≫80) for the Hα element compared to helium. This behavior may be attributed to mass exchange processes in FS CMa systems accompanied by an influx of hydrogen.

The information–entropy characteristics of complex stellar objects of the FS CMa type, $\tilde{I}$ and $\tilde{H}$, obtained from Formulas (9) and (15), are summarized in Table 2. In all cases, alternating transitions between the values of information and entropy are observed near their theoretical intersection point. These mutual transitions describe the system’s passage between states of “order” and “chaos”. For FS CMa-type stars, such transitions may reflect evolutionary stages and indicate the onset of active processes in their atmospheres or envelopes, as well as chaotic behavior of the system associated with bursts of activity, accretion, or matter ejection.

The application of the new characteristic $\tilde{E}\left(\lambda_{0},\lambda_{i}\right)$ defined by Formula (15) and accounting for deviations from the main spectral lines $\lambda_{0}$ with asymmetrically shifted wavelengths, demonstrates the potential of a novel approach to identifying and characterizing the state of binary stars, complementing existing methods. As shown in Table 2, for the characteristic $\tilde{I}\left(Y|X\right)$, its quantitative scale invariance values $I_{*}\left(p\right)=0.567,H\left(I_{*}\right)=0.806$ provide an unambiguous criterion for determining the evolutionary status of previously unclassified objects. The uncertainty in the classification of stars Nos. 6, 11, and 15 in Table 1 has thus been resolved—all these stars belong to evolutionary status II.

**Figure 10:**
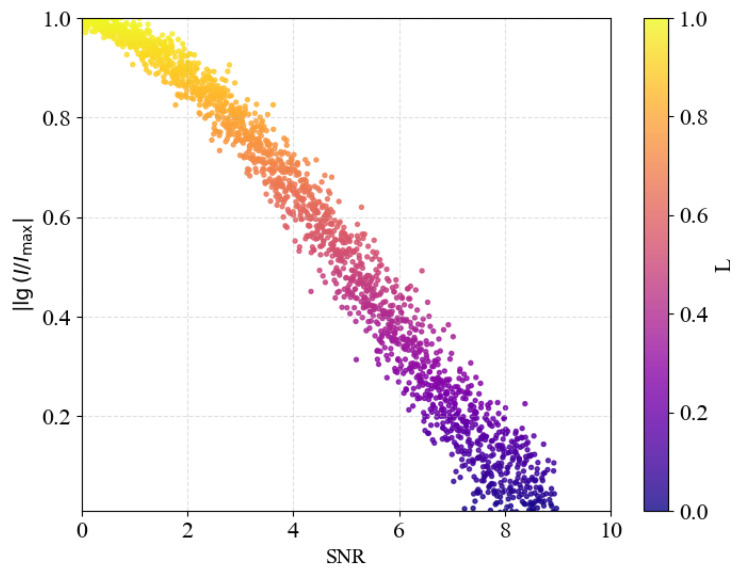
Illustrates the conditional information entropy on the Hertzsprung–Russell diagram as a function of the signal-to-noise ratio (SNR).

The potential for classifying stellar characteristics using conditional information is revealed through the differences between phase-space entropy and conditional entropy, with the goal of reproducing the characteristic structure of the well-known Hertzsprung–Russell (HR) diagram [42]. Figure 10 shows the transformation of Figure 1 into new coordinates,

$$\left|\frac{\mathrm{lg}(I(\mathrm{SNR}))}{I_{\max}}\right|,\;\mathrm{SNR}=\frac{1}{\tilde{H}\left(Y|X\right)}-1$$

in accordance with Equation (14) for the signal-to-noise ratio. According to modern observations, the maximum stellar luminosities on the HR diagram reach values of approximately ∼10^6^ [42]. For this reason, the logarithmic ratio of the absolute value of *I* is used for direct comparison with Figure 1.

## 4. Conclusions

Based on the results of the proposed methods for determining the evolutionary status of stars, the following conclusions can be drawn.

The theoretically derived criteria for scale-invariant, self-similar values of conditional information $\tilde{I}\left(Y|X,\lambda_{0}\right)$ make it possible to distinguish three principal regimes of stellar evolution: stochastic ($0.806\leq \tilde{I}<1$), chaotic ($0.567\leq \tilde{I}<0.806$), and transitional ($0.5\leq \tilde{I}<0.567$), ($0.433\leq \tilde{I}<0.5$). Complex stellar systems of the FS CMa type can also be classified according to these informational regimes using the normalized spectral intensity $\tilde{E}\left(\lambda_{0},\lambda_{i}\right)$, which accounts for asymmetries in the spectral profile.

Using a new characteristic—normalized intensity with an asymmetrically shifted wavelength of the two components of a binary system—a fundamental difference in the evolutionary status of FS CMa objects, most of which are binary stars, has been quantitatively established. This distinction reflects the presence of intense dust–gas fluctuations within the envelopes of binary systems. Moreover, a quantitative resolution was provided for the previously uncertain evolutionary classification (without considering the asymmetry of the information itself) of Aldebaran (II ÷ III), α Virginis (II), and Vega (II) listed in Table 2. Among these, one object demonstrates, both conversationally and theoretically, a transitional evolutionary status (II).

The proposed information–entropy framework reproduces the characteristic structure of the Hertzsprung–Russell (HR) diagram (Figure 1 and Figure 10). Massive, high-temperature objects exhibit luminosities close to the maximum value $\left(I∼I_{\max}\right)$ at minimal signal-to-noise ratios. The main sequence, represented by

$$\frac{\mathrm{lg}\left(I(\mathrm{SNR})\right)}{I_{\max}}∼\frac{1}{2},$$

corresponds to the transition domain between information and entropy. Beyond this region, a conditional decrease in entropy and a simultaneous increase in the information content of the signal-to-noise ratio are observed, accompanied by a gradual decline in temperature.

A more detailed description of the fluctuation structure of the HR diagram can be obtained by considering the difference between the fractal (*D*) and topological (*d*) dimensions, defined as

γ=D−d.

Within this framework, the condition D−d≲1 ensures probabilistic scale invariance in the interval 0<γ<1. TBurst-like features on the HR diagram correspond to regions where γ≳1, for example at D≳2,d=1, D≳3,d=2.

## Figures and Tables

**Table 1:** Spectral Class.

No.	Names of Stars	Temperature	Weight	Spectral Class	Evolutionary Status of a Star $\tilde{I}\left(Y|X,\lambda_{0}\right)$	Lit. Source
1	HD46223	45,316 K	$61.78\;M_{⊙}$	O4V	I	[12]
2	HD46150	43,181 K	$42.71\;M_{⊙}$	O5V	I	[12]
3	HD210839	36,000 K	$51.4\;M_{⊙}$	O	I	[13]
4	HD164492	33,600 K	$34\;M_{⊙}$	O7V	I	[14]
5	β Cephei (β Cephei)	27,000 K	$13.3\;M_{⊙}$	B2III	II	[15]
6	Spica (α Vir) primary	25,300 ± 500 K	$11.43\pm 1.15\;M_{⊙}$	B1V	II and III	[16]
7	Rigel (bet Ori)	12,130 ± 530 K	$18\;M_{⊙}$ 2.713±0.01mas	B8Ia	II	[17]
8	HD179218	10,400 ± 600 K	$2.9\;M_{⊙}$	B9	I	[18]
9	αCMa (Sirius)	10,500 K	$2.02\;M_{⊙}$	A0m	II	[19]
10	HD190073	9250±250K	$2.9\pm 0.5\;M_{⊙}$	A2e	II	[20]
11	Vega (α Lyrae)	9550±125K	$2.135\pm 0.074\;M_{⊙}$	A0V	II and III	[21]
12	HD35187	8000 K	$2.5M_{⊙}$	A7V	II	[22]
13	γ Cyg (γ Cygni)	5790 K	$14.5\;M_{⊙}$	F8 lab	II	[23]
14	α Aur (a Capella)	4940 K	$2.69\;M_{⊙}$	K0III	II	[24]
15	Aldebaran	4300 K	$1.13\pm 0.11\;M_{⊙}$	K5 III	II and III	[25]
16	M(μ) Cephei	3700 K	40–50 $M_{⊙}$	M2Ia	III	[26]
17	α Sco (α Scorpii)	3660 K	$12M_{⊙}$	M1.5lab	III	[27]
18	Betelgeuse (α Orionis)	3500 K	15–20 $M_{⊙}$	M1-2 Ia	III	[28]
19	o Cet (Peace)	2900 K	$1.2M_{⊙}$	M7III	III	[29]
20	CD-315070	12,500 ± 500 K	$20\;M_{⊙}$	B7-B8	II	[30]
21	MWC645	18,000 ± 2000 K	$7\;M_{⊙}$	B	III	[31]
22	MWC728	14,000 ± 1000 K	$2.3\times 10^{-2}\;M_{⊙}$	B5	III	[32]
23	BD+23 3183	9350±400K	$4\;M_{⊙}$	A0V	II	[33]
24	3Pup	8500±500K	$8.8\pm 0.5\;M_{⊙}$	A2.7Ib	II	[34]

**Table 2:** Evolutionary Status.

Evolutionary Status of a Star	Stars	$\tilde{I}\left(Y|X,<\left(\lambda_{0}\pm \Delta \lambda \right)>\right)$
I	HD210839	0.894
I	HD46223	0.927
I	HD46150	0.921
II	Rigel	0.617
II	HD35187	0.656
II	HD164492	0.701
II	Vega III	0.597
II	HD190073	0.649
II	Aldebaran III	0.650
II	α Vir (α Virginis) II	0.653
II	o Cet (Peace)	0.652
II	HD179218	0.649
II	β Cephei (β Cephei)	0.648
II	αCMa (Sirius)	0.655
II FS CMa	BD+23 3183	0.619
II FS CMa	CD-315070	0.640
III	α Sco (Antares)	0.382
III	α Aur (α Capella)	0.503
III	Betelgeuse	0.538
III	μ Cephei	0.568
III	γ Cyg (γ Cygni)	0.272
III FS CMa	MWC728	0.567
III FS CMa	3Pup	0.527
III FS CMa	MWC645	0.478

## Data Availability

The original contributions presented in this study are included in the article. Further inquiries can be directed to the corresponding author.

## Abbreviations

The following abbreviations are used in this manuscript:

FS CMa	B[e]-type stars of the FS Canis Majoris type
PMS	Pre-Main Sequence
MS	Main Sequence
